# Molecular Epidemiology of Carbapenem-Resistant *Pseudomonas aeruginosa* Before the COVID-19 Pandemic: Resistance Profiles and Clonality in a Tertiary-Care Hospital

**DOI:** 10.3390/antibiotics15010102

**Published:** 2026-01-20

**Authors:** Raúl Eduardo Loredo-Puerta, Perla Niño-Moreno, Raúl Alejandro Atriano-Briano, Katy Lizbeth Martínez-Alaniz, Nubia Baltazar-Benitez, Luis Fernando Pérez-González, Mónica Lucía Acebo-Martínez, Adriana Berenice Rousset-Román, Edgar A. Turrubiartes-Martínez

**Affiliations:** 1 Faculty of Chemical Sciences, Autonomous University of San Luis Potosí, Av. Dr. Manuel Nava 6, Zona Universitaria, San Luis Potosí 78210, Mexico; raul.loredo@cinvestav.mx (R.E.L.-P.); ncarmenp@uaslp.mx (P.N.-M.); raul.atriano@uaslp.mx (R.A.A.-B.); nubia.baltazar@uaslp.mx (N.B.-B.); 2 Department of Genetics and Molecular Biology, Center for Research and Advanced Studies of the National Polytechnic Institute, Av. Instituto Politécnico Nacional 2508, Col. San Pedro Zacatenco, Ciudad de México 07360, Mexico; 3 Clinical Laboratory, Dalinde Medical Center, Tuxpan 25, Col. Roma, Ciudad de México 06760, Mexico; 4 Center for Research in Health Sciences and Biomedicine, Autonomous University of San Luis Potosí, Av. Sierra Leona 550, Col. Lomas de San Luis, San Luis Potosí 78210, Mexico; 5 Innovation Unit for Clinical Laboratory Sciences, Autonomous University of San Luis Potosí, Av. Dr. Manuel Nava 6, Zona Universitaria, San Luis Potosí 78210, Mexico; katy.martinez@uaslp.mx; 6 Faculty of Nursing and Nutrition, Autonomous University of San Luis Potosí, Niño Artillero 150, Zona Universitaria, San Luis Potosí 78240, Mexico; monica.acebo@uaslp.mx (M.L.A.-M.); adriana.rousset@uaslp.mx (A.B.R.-R.); 7 Department of Infectious Diseases, Regional High Specialty Hospital “Dr. Ignacio Morones Prieto”, Av. Venustiano Carranza 2395, San Luis Potosí 78290, Mexico; fernando.perez@uaslp.mx

**Keywords:** *Pseudomonas aeruginosa*, carbapenem-resistant, carbapenemase, multilocus sequence typing, healthcare-associated infection, intensive care unit, *bla*_GES_, *bla*_IMP_, *bla*_VIM_, ST309, ST411, ST167

## Abstract

**Background/Objectives**: *Pseudomonas aeruginosa* is an opportunistic pathogen frequently implicated in healthcare-associated infections, particularly ventilator-associated pneumonia and other device-related infections. The global emergence of carbapenem-resistant *P. aeruginosa* (CRPA) represents a major clinical challenge due to its limited therapeutic options and high mortality rates. **Methods**: Relevant clinical data were obtained from medical records. Isolates were identified via 16S PCR, and antimicrobial susceptibility testing was performed using the Vitek2 Compact system following CLSI guidelines. Carbapenemase genes (blaGES, blaKPC, blaIMP, blaNDM, blaVIM) were detected via PCR. Clonal relationships were determined via RAPD-PCR, and some sequence types were assigned according to the global *P. aeruginosa* MLST database. **Results**: In this study, 40 non-duplicate CRPA isolates were collected from 35 patients in a tertiary-care hospital in Mexico. Most isolates originated from adult patients, predominantly from tracheal aspirates (32.5%) and urine cultures (25.0%). Mechanical ventilation was the most common invasive device associated with infection, and the overall mortality rate reached 14.3%. Antimicrobial susceptibility testing showed that 95% of isolates exhibited a multidrug-resistant phenotype, with high resistance rates to ciprofloxacin (70.0%) and β-lactams. Carbapenemase genes were detected in 55% of isolates, mainly *bla*_IMP_, *bla*_GES_, and *bla*_VIM_, either alone or in combination. Notably, this is the first report of ST309 (*bla*_IMP_), ST411 (*bla*_GES_ + *bla*_IMP_), and ST167 (*bla*_GES_ + *bla*_VIM_) carrying carbapenemase genes in Mexico. **Conclusions**: These findings highlight the persistence and genetic diversity of CRPA circulating in hospital settings and emphasize the urgent need for strengthened genomic surveillance and infection control programs to prevent the spread of these high-risk multidrug-resistant clones.

## 1. Introduction

*Pseudomonas aeruginosa* is a non-fermenting, opportunistic, aerobic Gram-negative bacillus that is widely recognized as a major causative agent of healthcare-associated infections (HAIs) [1,2]. Its remarkable ability to adapt to harsh environmental conditions enables it to persist and disseminate in hospital environments, particularly on moist surfaces, medical devices, and in antiseptic solutions [3]. This pathogen is especially relevant in intensive care units (ICUs), where it ranks among the leading causes of ventilator-associated pneumonia (VAP), a condition associated with high mortality rates [4,5]. In addition, it is responsible for catheter-associated urinary tract infections, bloodstream infections, and surgical site and burn wound infections, particularly in immunocompromised patients, in whom it can lead to sepsis [6,7]. A major concern surrounding *P. aeruginosa* is the emergence of carbapenem-resistant strains (carbapenem-resistant *P. aeruginosa*, CRPA) [8]. In 2017, the World Health Organization (WHO) classified CRPA as a critical priority pathogen for research and development of new antibiotics due to the scarcity of effective treatment options [9]. In its 2024 updated list, the WHO reclassified CRPA as a high-priority pathogen, reflecting declining resistance rates observed in some countries and emergence of new therapeutic options. Nevertheless, the global threat remains significant, particularly in regions with limited access to effective antimicrobials, insufficient diagnostic infrastructure, inadequate knowledge of local microbial epidemiology, and underdeveloped antimicrobial stewardship programs [10].

CRPA infections are particularly difficult to treat due to the presence of multiple resistance mechanisms. These include the production of carbapenemases—such as metallo-β-lactamases (IMP, VIM, NDM) and serine β-lactamases (KPC)—loss of porins (notably OprD), overexpression of efflux pumps (e.g., MexAB-OprM), and biofilm formation. Genes encoding these resistance determinants are often located on mobile genetic elements, which facilitate horizontal gene transfer among bacterial species and contribute to the rapid spread of antimicrobial resistance [5,8,11]. Of particular concern is the emergence of high-risk clones, including ST235, ST111, ST175, and ST244, which are widely distributed globally and have been associated with nosocomial outbreaks. These clones are characterized by multidrug resistance, increased virulence, and an enhanced ability to evade both therapeutic interventions and infection control measures [12,13,14]. Their presence in HAIs represents a significant public health challenge, especially in low- and middle-income countries, where the availability of last-resort antibiotics is limited, and surveillance and stewardship infrastructures are often inadequate [15,16].

In Mexico, national strategies such as the Hospital Epidemiological Surveillance Network (RHOVE), and academic networks like the University Program of Health Research (PUIS) from the Universidad Nacional Autónoma de México (UNAM) through its PUCRA network, along with the Network for the Research and Surveillance of Drug Resistance (INVIFAR), have been implemented to monitor and control the spread of multidrug-resistant pathogens, aiming to reduce morbidity, mortality, and hospital length of stay. However, there is still a critical need to strengthen local epidemiological surveillance of resistant *P. aeruginosa* to support evidence-based prevention and treatment strategies [17,18]. It is of vital importance to reinforce this surveillance before the emergence of new respiratory viruses capable of causing pneumonia. In this context, the molecular and clonal characterization of CRPA in tertiary healthcare settings is essential for optimizing clinical management [18,19]. The analysis of microbial genomes allows the identification of specific resistance genes, which facilitates the prediction of antibiotic efficacy against particular strains. In the case of CRPA, the presence of carbapenemases is a key determinant of resistance to this important class of antibiotics, making their detection essential for guiding therapeutic decisions [20]. Furthermore, clonality studies provide valuable epidemiological information, enabling the identification of dissemination patterns and supporting the implementation of more effective infection control strategies [21].

The objective of this study was to describe the resistance patterns of CRPA to various antibiotics, identify the predominant carbapenemase-encoding genes, and characterize the distribution of high-risk clones in a tertiary-care Mexican hospital.

## 2. Results

### 2.1. Clinical Isolates

We included 40 non-duplicate CRPA isolates from 35 patients, each obtained from a distinct clinical specimen. CRPA isolates were predominantly recovered from patients in the adult age group (36–64 years; 48.6%), followed by young adults (18–35 years; 28.6%). The average hospital stay before the isolation of CRPA was 27.9 days, while after isolation it was 16.7 days. The mortality rate in the patients of this study was 14.3%, corresponding to 5 deaths, 4 of which were associated with pneumonia, urinary tract infections, bacteremia, and soft tissue infections, respectively. Twenty patients received one or more prior antimicrobial therapies, with carbapenem antibiotics being the most commonly used (31.4%), followed by glycopeptides (20.0%) and quinolones (14.3%) (Table 1).

Tracheal aspirate was the most frequent specimen type among all isolates (32.5%), followed by urine cultures (25.0%). The clinical isolates originated from various hospital departments, predominantly from the male surgery ward, followed by the intensive care unit (ICU) (24.5%). The most frequently used medical device associated with these infections was mechanical ventilation (30.0%) (Table 2). Pneumonia and urinary tract infections together accounted for over half of the CRPA-related infections.

We included 40 non-duplicate isolates of CRPA from 35 patients. Most of the CRPA isolates came from adult patients (48.6%). The average hospital stay before the isolation of CRPA was 27.9 days, while after isolation it was 16.7 days. The mortality rate in the patients of this study was 14.3%, corresponding to five deaths, four of which were associated with pneumonia, urinary tract infections, bacteremia, and soft tissue infections, respectively. Twenty patients received one or more prior antimicrobial therapies, with carbapenem antibiotics being the most used (31.4%), followed by glycopeptides (20.0%) and quinolones (14.3%) (Table 1).

### 2.2. Antibiotic Susceptibility Pattern

**Figure 1:**
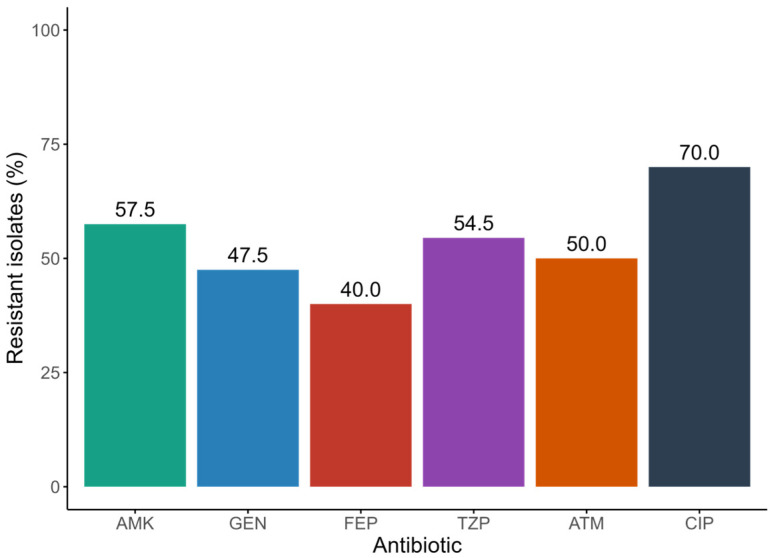
Resistance percentages to different antibiotics of CRPA strains. AMK, amikacin; GEN, gentamicin; FEP, cefepime; TZP, piperacillin/tazobactam; ATM, aztreonam; CIP, ciprofloxacin.

**Figure 2:**
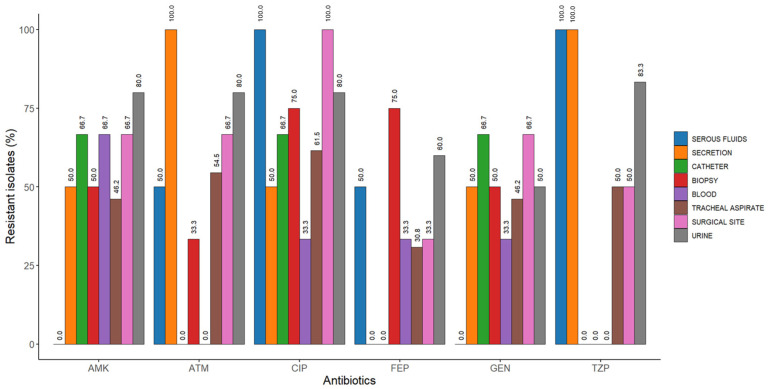
Resistance percentages by specimen type to different antibiotics of CRPA strains. AMK, amikacin; ATM, aztreonam; CIP, ciprofloxacin; FEP, cefepime; GEN, gentamicin; TZP, piperacillin/tazobactam.

**Figure 3:**
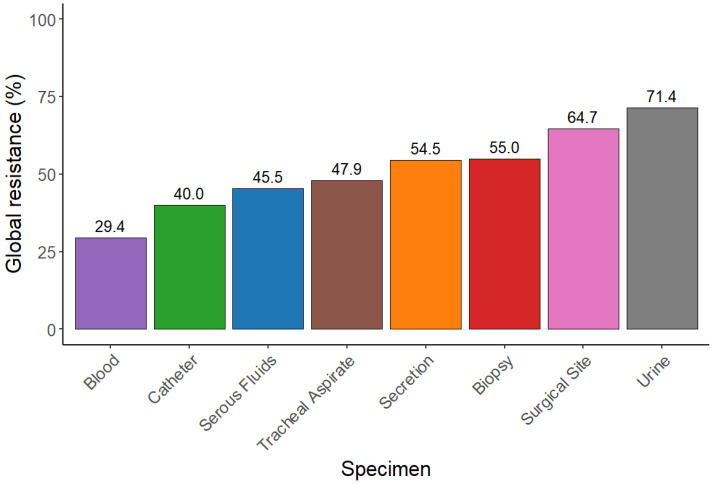
Global resistance percentages by specimen type for CRPA strains.

The CRPA strains included in the study, in addition to resistance to meropenem, exhibited resistance rates to other antimicrobials greater than 40%, with the primary antibiotic being ciprofloxacin (70.0%) (Figure 1 and Table 3). Among the 40 CRPA isolates analyzed, 38 (95.0%) displayed a multidrug-resistant (MDR) profile. The most frequent resistance phenotype identified was amikacin, gentamicin, ciprofloxacin, and meropenem, which was detected in six isolates (Table 3 and Figure 2). The urine specimen showed the highest resistance to the antibiotics analyzed in this study (Figure 2 and Figure 3).

### 2.3. Presence of Genes Encoding Carbapenemases

**Figure 4:**
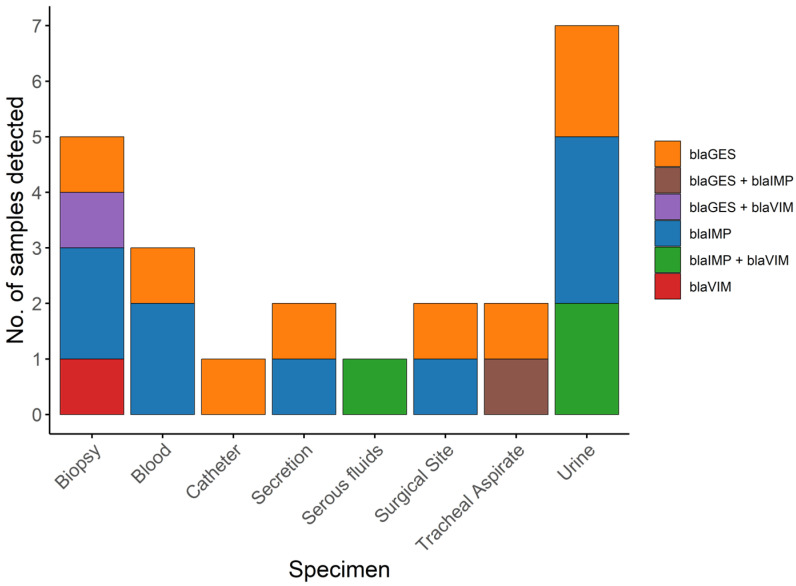
Number of isolates with detected carbapenemases by specimen type for CRPA strains.

**Figure 5:**
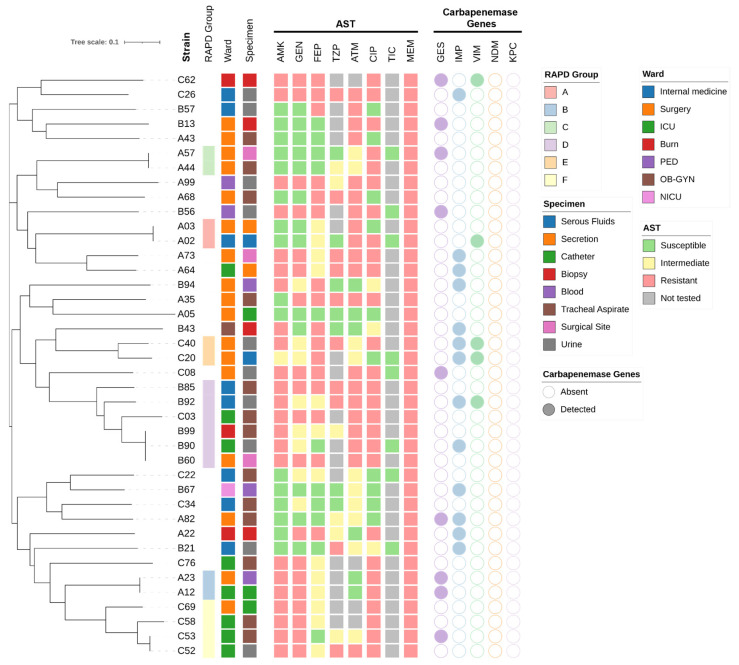
Dendrogram showing clonal groups of CRPA strains based on RAPD-PCR analysis. The figure also displays relevant metadata for each isolate, including specimen type, antibiotic resistance profiles, and the presence or absence of resistance genes. AST, Antimicrobial Susceptibility Testing; AMK, amikacin; GEN, gentamicin; FEP, cefepime; TZP, piperacillin/tazobactam; ATM, aztreonam; CIP, ciprofloxacin; TIC, Ticarcillin; MEM, Meropenem.

More than half of the isolates (*n* = 22) had a gene encoding a carbapenemase, and their distribution was as follows: 9 isolates carried *bla*_IMP_ (22.5%), 7 carried *bla*_GES_ (17.5%), and one carried *bla*_VIM_ (2.5%). Additionally, in three isolates, the genes coding for the carbapenemases *bla*_IMP_ and *bla*_VIM_ were detected concomitantly (7.5%), in one isolate, *bla*_IMP_ and *bla*_GES_ (2.5%), and in another, *bla*_VIM_ and *bla*_GES_ genes (2.5%, 1); among the urine specimens, carbapenemases were detected in 7 out of 10 isolates, with one or two enzymes present per isolate. The next most frequently affected specimen type was biopsy samples (see Figure 4 and Figure 5).

### 2.4. Clonal Diversity and Molecular Epidemiology of CRPA Isolates

Six distinct clonal groups (A–F) were identified based on RAPD banding patterns. Six isolates belonged to group D, four to group F, and groups A, B, C, and E were each represented by two isolates (see Figure 2). Clonal groups affected the Surgery and Internal Medicine wards; however, one clonal group (D) was detected across four different hospital departments, while another clonal group (F) was mainly distributed in the ICU (see Figure 2). Six strains were selected based on their resistance profile and carbapenemase production. Through MLST analysis, it was determined that strains A57, A64, and A73 corresponded to ST309, A82 and B67 to ST411, and C62 to ST167

## 3. Discussion

In this study, 40 non-duplicate carbapenem-resistant *Pseudomonas aeruginosa* (CRPA) isolates from 35 patients—mostly adults—were analyzed, with a mortality rate of 14.3%. In the hospital setting, *P. aeruginosa* remains one of the most relevant pathogens associated with healthcare-associated infections, particularly those linked to the use of invasive medical devices such as mechanical ventilation. This intervention, frequently required in critically ill patients—including those with COVID-19—represents a well-established risk factor for the acquisition of multidrug-resistant strains [22,23].

Fatal outcomes in this cohort were primarily associated with ventilator-associated pneumonia, as well as catheter-associated urinary tract infections, bloodstream infections, and soft-tissue infections following surgical procedures. Three of these deaths were caused by CRPA isolates exhibiting a multidrug-resistant (MDR) carbapenemase-producing phenotype, underscoring the significant clinical impact of these strains in hospital environments.

Prior and prolonged exposure to β-lactam antibiotics is a well-documented risk factor contributing to the selection of carbapenem-resistant strains. In our cohort, 40% of patients with CRPA had previously received β-lactam therapy, and 95% of isolates displayed an MDR phenotype. These findings reinforce the hypothesis that selective antibiotic pressure drives the emergence and persistence of CRPA in healthcare settings [24].

From a genetic standpoint, more than half of the isolates harbored carbapenemase-encoding genes, with *bla*_IMP_ (32.5%), *bla*_GES_ (22.5%), and *bla*_VIM_ (12.5%) being the most prevalent. These frequencies exceed those reported in previous studies, such as Garza-González et al. (2021), where rates of these determinants were 25.3%, 13.1%, and 13.1%, respectively, in a multicenter survey across 47 Mexican hospitals [25]. Notably, 45% of isolates did not carry any of the carbapenemase genes evaluated, suggesting the involvement of alternative resistance mechanisms such as efflux pump overexpression, porin loss, or the presence of undetected carbapenemases [26].

Clonal analysis revealed six distinct genetic groups, demonstrating substantial diversity among circulating strains. High-risk clones ST309, ST411, and ST167 were identified, all of which are recognized for their enhanced virulence, antimicrobial resistance, and epidemic potential, representing a serious global public health concern [27]. The ST309 clone, carrying *bla*_GES_ and *bla*_IMP_, previously reported in Mexico by Morales-Espinosa et al. (2017) as a cause of pediatric bacteremia, was also detected here, indicating the sustained circulation of this high-risk lineage in the country [28]. Importantly, this report constitutes the first detection in Mexico of ST411 (*bla*_GES_ + *bla*_IMP_) and ST167 (*bla*_GES_ + *bla*_VIM_) in isolates associated with HAIs, emphasizing the relevance of genomic surveillance programs for the early identification of emerging and high-risk clones.

Epidemiologically, CRPA infections occurred most frequently in surgical wards and intensive care units, where the routine use of invasive devices such as mechanical ventilation is common. This highlights the critical role of these devices in facilitating CRPA transmission and increasing infection risk among critically ill patients, including those with SARS-CoV-2 coinfection.

Overall, our findings highlight the urgent need to strengthen infection prevention and control (IPC) strategies and hospital-based surveillance programs to limit the dissemination of multidrug-resistant (MDR) Pseudomonas aeruginosa. The high prevalence of antimicrobial resistance, together with the co-circulation of multiple clones across different clinical areas, substantially increases the risk of nosocomial outbreaks and further restricts available therapeutic options. Recent evidence indicates that sustained and rigorously implemented IPC measures are critical to reducing the transmission of carbapenem-resistant *P. aeruginosa* in healthcare settings [29,30]. In parallel, active microbiological surveillance supported by molecular epidemiology facilitates early identification of transmission events, enabling timely targeted interventions and effective outbreak containment [31].

In addition, the rational use of last-resort antibiotics, supported by antimicrobial stewardship programs (ASPs), remains essential to reduce selective pressure and delay the emergence of further resistance. Contemporary studies demonstrate that hospital-based ASPs are associated with a reduction in MDR *P. aeruginosa* incidence and improved clinical outcomes [32]. Furthermore, the evaluation of combination antimicrobial therapies guided by pharmacodynamic principles may optimize the management of carbapenem-resistant *P. aeruginosa* (CRPA) infections, particularly in critically ill patients or those coinfected with SARS-CoV-2, in whom disease severity and prolonged hospitalization further increase the risk of infection and transmission [33]. Collectively, the integration of robust IPC practices, active surveillance, and optimized antimicrobial use represents a comprehensive approach to reducing the burden of MDR *P. aeruginosa* and preventing future nosocomial outbreaks.

This study has some limitations, as it was conducted in a single tertiary-care hospital in Mexico over a 13-month period, which may limit generalizability. MICs could not be determined for all antibiotics, and only the most common carbapenemase genes were analyzed, so other resistance mechanisms may have been missed. Clonal relationships were assessed using RAPD-PCR and MLST without whole-genome sequencing, limiting the resolution of the analysis. Multicenter studies across diverse regions of the country are warranted to obtain a more comprehensive understanding of the prevalence and distribution of CRPA in nationwide healthcare facilities. Additionally, broader resistome analyses and the assessment of novel therapeutic strategies are recommended to enhance clinical outcomes and improve preparedness for emerging threats, such as those experienced during the COVID-19 pandemic.

## 4. Materials and Methods

### 4.1. Bacterial Isolates and Clinical Data

*Pseudomonas aeruginosa* isolates associated with HAIs were collected at the Hospital Central “Dr. Ignacio Morones Prieto” located in San Luis Potosí, Mexico, during the period from May 2018 to June 2019. Clinical specimens were collected and processed in accordance with the recommendations of the Clinical and Laboratory Standards Institute (CLSI) [34,35,36,37]. A total of 40 non-duplicate *P. aeruginosa* isolates were included, all of which exhibited a minimum inhibitory concentration (MIC) ≥8.0 µg/mL to at least one carbapenem antibiotic. Relevant clinical data were collected from the medical records, including patient demographic information, name of the hospital ward of origin, admission and discharge diagnoses, use of medical devices, prior antibiotic therapies before the isolation of CRPA, as well as the number of hospital days before and after sample collection.

### 4.2. Identification and Antimicrobial Susceptibility Testing

Identification and antibiogram of all CRPA isolates were performed using the Vitek^®^2 system (BioMérieux SA, F-69280, Marcy l’Etoile, France) with GN NR. 21341 Card for the following antibiotics: Amikacin (AMK), Gentamicin (GEN), Cefepime (FEP), Piperacillin-Tazobactam (TZP), Aztreonam (ATM), Ciprofloxacin (CIP), Meropenem (MEM), Ticarcillin (TIC). The antimicrobial susceptibility of the strains was determined according to the guidelines and breakpoints of CLSI, M100-ED28:2018 [38]. MICs were not determined for all isolates due to the limited availability of antibiotics in the hospital. All isolates were successfully subcultured, and pure colonies were isolated for DNA extraction. The genus and species were confirmed by 16S PCR using primers and conditions previously reported [39].

### 4.3. Detection of Resistance Genes

Resistance genes encoding the following carbapenemases were identified: *bla*_GES_, *bla*_KPC_, *bla*_IMP_, *bla*_NDM_, *bla*_VIM_. The oligonucleotide sequences used for carbapenemase detection (Supplementary Table S1) were obtained from previously published studies [26,40].

### 4.4. Clonal Relationship and Molecular Typing

To determine the clonal relationship among CRPA isolates, the RAPD-PCR technique was performed using oligonucleotide 272-AGCGGGCCAA, and confirmed with oligonucleotide 208-ACGGCCGACC, as previously described [41,42]. For the analysis of RAPD products, PCR amplicons were resolved on 1.5% (*w*/*v*) agarose gels using TBE 1× buffer, and product sizes were estimated with 1 kb DNA size markers. Similarity among isolates was evaluated using the Jaccard similarity coefficient, and a dendrogram was constructed using the Neighbor-Joining algorithm, with analyses performed in RStudio using the vegan and ape packages. Only the main reproducible bands with consistent intensity were considered for the similarity matrix, and a cut-off value of ≥80% was applied to define clonal relatedness. The resulting tree was visualized and annotated using the Interactive Tree Of Life (iTOL) tool v6 (https://itol.embl.de, accessed on 1 May 2025) [43].

Molecular typing was conducted using the *Pseudomonas aeruginosa* MLST scheme developed by Curran et al. (2004), targeting the following housekeeping genes: *acsA*, *aroE*, *guaA*, *mutL*, *nuoD*, *ppsA*, and *trpE* [44]. Each gene was amplified via PCR and sequenced using the ABI 3130 Genetic Analyzer (Applied Biosystems, Waltham, MA, USA). Gene sequences were aligned with the global *Pseudomonas aeruginosa* MLST database (https://pubmlst.org/organisms/pseudomonas-aeruginosa, accessed on 1 May 2025) to determine the corresponding alleles, and sequence types (STs) were assigned based on the allelic profiles.

### 4.5. Statistical Analysis

The data were entered into a spreadsheet and analyzed using the tydyverse package of RStudio software (2025.05.1+513). The results of the analysis were displayed in frequency tables.

## 5. Conclusions

This study highlights the alarming presence of multidrug-resistant and carbapenemase-producing *Pseudomonas aeruginosa* in a tertiary-care hospital in Mexico. The detection of blaIMP, blaGES, and blaVIM genes, along with the identification of high-risk clones ST309, ST411, and ST167 reported for the first time in Mexico with carbapenemase genes, demonstrates the ongoing evolution and spread of resistance. These findings underscore the urgent need to strengthen infection control, antimicrobial stewardship, and genomic surveillance programs to limit dissemination and guide effective therapeutic strategies against high-risk CRPA clones in hospital settings.

## Figures and Tables

**Table 1:** Epidemiological characteristics of patients with CRPA isolates from HAIs in a tertiary-care hospital in Mexico, from May 2018 to June 2019.

Characteristic	Patients *n* = 35	(%)
**Age Groups (years)**		
Early Childhood (0–5)	1	(2.9)
Childhood (6–11)	1	(2.9)
Adolescence (12–17)	3	(8.6)
Young Adult (18–35)	10	(28.6)
Adult (36–64)	17	(48.6)
Elderly (>64)	3	(8.6)
**Sex**		
Male	24	(68.6)
Female	11	(31.4)
**Mortality**		
Associated	4	(11.4)
Not Associated	1	(2.8)
**Prior Antimicrobial Therapies**		
With β-lactams	14	(40.0)
With non- β-lactam antibiotics	6	(17.1)

**Table 2:** Microbiological characteristics of CRPA isolates derived from HAIs in a tertiary-care hospital in Mexico, from May 2018 to June 2019.

Characteristic	CRPA *n* = 40	(%)
**Type of culture**		
Tracheal aspirate	13	(32.5)
Biopsy	4	(10.0)
Catheter	3	(7.5)
Blood culture	3	(7.5)
Serous fluids	2	(5.0)
Secretion	2	(5.0)
Surgical site	3	(7.5)
Urine culture	10	(25.0)
**Hospital Service**		
Surgery	12	(42.5)
Gynecology	1	(2.5)
Internal Medicine	9	(22.5)
Burn Unit	2	(5.0)
Intensive Care Unit	9	(22.5)
Neonatal Intensive Care Unit	1	(2.5)
Pediatric	1	(2.5)
Use of medical devices *	25	(62.5)
Central venous catheter	5	(12.5)
Urinary catheter	7	(17.5)
Mechanical ventilation	12	(30.0)
Tracheal tube	1	(2.5)

* Use within the last 48 h, associated with the type of culture.

**Table 3:** Minimum inhibitory concentration of 40 non-duplicate CRPA isolates derived from HAIs in a tertiary-care hospital in Mexico.

Strain	MIC (µg/mL)							
	AMK	GEN	FEP	TZP	ATM	CIP	MEM	TIC
A02	≤2	≤1	16	≥128	≥64	≥4	≥16	4
A03	≤2	≤1	16	ND	32	≤0.25	8	ND
A05	≤2	≤1	≤1	8	4	≤0.25	8	ND
A12	≥64	≥16	16	ND	4	≥4	≥16	ND
A22	16	≥16	≥64	32	4	2	≥16	ND
A23	≥64	≥16	16	ND	4	≥4	≥16	ND
A35	8	≥16	32	≥128	32	≥4	≥16	ND
A43	4	≤1	4	ND	32	0.5	≥16	ND
A44	16	4	8	32	16	≥4	≥16	ND
A57	16	4	4	16	16	≥4	≥16	≥8
A64	≥64	≥16	16	≥128	≥64	≥4	≥16	ND
A68	≤2	≤1	≥64	≥128	≥64	0.5	≥16	ND
A73	≥64	≥16	16	≥128	≥64	≥4	≥16	ND
A82	≤2	≤1	4	64	16	0.5	≥16	ND
A99	≥64	≥16	≥64	32	≥64	≥4	≥16	ND
B13	≤2	≤1	8	ND	32	2	≥16	ND
B21	8	≤1	4	≥128	16	1	≥16	2
B43	≥64	≤1	≥64	ND	2	1	≥16	ND
B56	≥64	≥16	≥64	ND	≥64	≥4	≥16	≥8
B57	≤2	2	≥64	ND	≥64	0.5	≥16	ND
B60	≥64	≥16	32	ND	32	≥4	≥16	ND
B67	≤2	2	2	8	8	≤0.25	8	ND
B85	≥64	≥16	32	≥128	≥64	≥4	≥16	ND
B90	≥64	8	8	ND	32	≥4	≥16	≥8
B92	≥64	8	16	≥128	≥64	≥4	≥16	ND
B94	≥64	8	≥64	8	2	1	8	ND
B99	≥64	8	16	64	32	≥4	≥16	ND
C03	≥64	≥16	≥64	ND	≥64	≥4	≥16	ND
C08	≥64	≥16	≥64	ND	≥64	≥4	≥16	≥8
C20	32	8	≥64	ND	16	2	≥16	≥8
C22	16	8	16	ND	16	≤0.25	≥16	≥8
C26	≥64	≥16	≥64	≥128	≥64	≥4	≥16	ND
C34	16	8	8	≥128	16	≤0.25	≥16	ND
C40	≥64	8	≥64	≥128	16	≥4	≥16	ND
C52	≥64	≥16	16	≥128	32	≥4	≥16	ND
C53	≥64	≥16	8	64	16	≥4	≥16	ND
C58	≥64	≥16	16	ND	ND	≥4	≥16	ND
C62	≥64	≥16	≥64	ND	ND	≥4	≥16	ND
C69	≥64	≥16	16	ND	ND	≥4	≥16	ND
C76	≥64	≥16	16	ND	ND	≥4	≥16	ND

ND: No data. AMK, amikacin; GEN, gentamicin; FEP, cefepime; TZP, piperacillin/tazobactam; ATM, aztreonam; CIP, ciprofloxacin; TIC, Ticarcillin; MEM, Meropenem.

## Data Availability

Data are contained within the article and Supplementary Materials.

## Supplementary Materials

The following supporting information can be downloaded at https://www.mdpi.com/article/10.3390/antibiotics15010102/s1. Table S1: Oligonucleotides used for carbapenemase detection in this study.

## Abbreviations

AST	Antimicrobial Susceptibility Testing
CRPA	Carbapenem-resistant Pseudomonas aeruginosa
ICU	Intensive Care Unit
HAIs	Healthcare-Associated Infections
MLST	Multilocus Sequence Typing
RAPD	Random Amplified Polymorphic DNA
ST	Sequence Type
