# Supplementary material for: Molecular Epidemiology of Carbapenem-Resistant Pseudomonas aeruginosa Before the COVID-19 Pandemic: Resistance Profiles and Clonality in a Tertiary-Care Hospital
Source: Antibiotics (Basel). 2026 Jan 20;15(1):102. doi: 10.3390/antibiotics15010102 (PMC12837621; doi:10.3390/antibiotics15010102)
Supplement: Supplementary file 1 [file antibiotics-15-00102-s001.zip › antibiotics-4043752-supplementary.pdf]

Table S1: Oligonucleotides used for carbapenemase detection in this study

ID	Sequence (5´-3´)	Target	Product size (pb)	Reference
ges-F	CTT CAT TCA CGC ACT ATT AC	*bla*GES	827	(40)
ges-R	TAA CTT GAC CGA CAG AGG			
imp-F	GGA ATA GAG TGG CTT AAY TCT C	*bla*IMP	232	(26)
imp-R	GGT TTA AYA AAA CAA CCA CC			
kpc-F	CGT CTA GTT CTG CTG TCT TG	*bla*KPC	798	(26)
kpc-R	CTT GTC ATC CTT GTT AGG CG			
ndm-F	GGT TTG GCG ATC TGG TTT TC	*bla*NDM	621	(40)
ndm-R	CGG AAT GGC TCA TCA CGA TC			
vim-F	GAT GGT GTT TGG TCG CAT A	*bla*VIM	390	(40)
vim-R	CGA ATG CGC AGC ACC AG			
